# Reconstructing the emergence of a lethal infectious disease of wildlife supports a key role for spread through translocations by humans

**DOI:** 10.1098/rspb.2016.0952

**Published:** 2016-09-28

**Authors:** Stephen J. Price, Trenton W. J. Garner, Andrew A. Cunningham, Tom E. S. Langton, Richard A. Nichols

**Affiliations:** 1 UCL Genetics Institute, University College London, Darwin Building, Gower Street, London WC1E 6BT, UK; 2 Institute of Zoology, Zoological Society of London, London NW1 4RY, UK; 3 School of Biological and Chemical Sciences, Queen Mary University of London, Mile End Road, London E1 4NS, UK; 4 Herpetofauna Consultants International, Triton House, Bramfield, Halesworth, Suffolk IP19 9AE, UK

**Keywords:** pathogen pollution, ranavirus, citizen science, wildlife disease, anthropogenic drivers, spatio-temporal models

## Abstract

There have been few reconstructions of wildlife disease emergences, despite their extensive impact on biodiversity and human health. This is in large part attributable to the lack of structured and robust spatio-temporal datasets. We overcame logistical problems of obtaining suitable information by using data from a citizen science project and formulating spatio-temporal models of the spread of a wildlife pathogen (genus *Ranavirus*, infecting amphibians). We evaluated three main hypotheses for the rapid increase in disease reports in the UK: that outbreaks were being reported more frequently, that climate change had altered the interaction between hosts and a previously widespread pathogen, and that disease was emerging due to spatial spread of a novel pathogen. Our analysis characterized localized spread from nearby ponds, consistent with amphibian dispersal, but also revealed a highly significant trend for elevated rates of additional outbreaks in localities with higher human population density—pointing to human activities in also spreading the virus. Phylogenetic analyses of pathogen genomes support the inference of at least two independent introductions into the UK. Together these results point strongly to humans repeatedly translocating ranaviruses into the UK from other countries and between UK ponds, and therefore suggest potential control measures.

## 1. Background

Emerging infectious diseases (EIDs) are defined as diseases undergoing an increase in incidence, geographical range, or host range. EIDs of humans, livestock, and crops are increasingly recognized as major challenges, because they can impose massive economic burdens and have major public health implications [1]. By contrast, much interest in wildlife diseases has been indirect, a consequence of wildlife populations serving as reservoirs for human diseases (zoonoses, see [2]) and diseases of livestock (e.g. bovine tuberculosis and rinderpest [3,4]). A second, more direct motivation for understanding wildlife EIDs is their impact on biodiversity, since they can cause extirpation and/or catastrophic multihost declines [5–9].

Preventing EIDs at source is highly desirable; but such intervention requires a thorough understanding of the drivers of emergence. Reconstruction of modes of transmission and patterns of spread can inform strategic management approaches. For example, Jennelle *et al.* [10] demonstrated how deer harvesting could be implemented to manage chronic wasting disease prevalence in deer populations. However, the analysis of the dynamics of wildlife disease spread and the application of molecular epidemiological techniques to investigate them are increasing only slowly [11].

One effective approach to reconstructing emergence is to use phylodynamic techniques [12]. These methods are most effective when genetic data are available for large samples that have been serially sampled at known locations and for pathogens with high mutation rates and large population sizes (such as fast-evolving viruses with RNA genomes). Suitable datasets are more frequently available for human diseases, those posing a zoonotic risk and those of economic importance. Unfortunately, for other diseases of wildlife, the required knowledge of pathogen diversity and host susceptibility is usually lacking and the genetic patterns may lack sufficient resolution [13]. Here, we develop an alternative approach to reconstruct pathogen spread and test hypotheses relating to drivers of emergence, which could be used as a model for other wildlife diseases. We demonstrate how citizen science can be employed to generate large datasets that feed spatio-temporal models of emergence, and how this approach can be integrated with the type of patchy genetic sampling that may be common to studies of wildlife diseases.

To study emergence of an infectious disease of amphibians (ranavirosis), we made use of an ongoing citizen science project in the UK that has collated records of amphibian mortality for two decades and has provided material for genetic characterization of the viruses responsible (genus *Ranavirus*). Ranaviruses are large, double-stranded DNA viruses that infect and cause severe disease in amphibians, reptiles, and fish on five continents [14]. In the UK, ranavirus infections and mass mortality events have been recorded and investigated since 1992 following alarm by members of the public [15]. Infections and reports of mortality have focused on *Rana temporaria* a species which has shown median virus-driven declines of 81% sustained over a 12-year period [15,16].

There is *prima facie* evidence linking ranavirus spread to humans in a number of ways. Ranaviruses have been found in traded amphibians [17], a number of outbreaks have been associated with introduced or farmed species [18–21], and an earlier study that used microscopy and molecular methods to compare viruses suggested that ranaviruses were introduced to the UK from North America [22]. Human activity has also been correlated with increased ranavirus prevalence in North America [23] and urbanization is correlated with ranavirus occurrence in the UK [24]. In North America, the use of infected juvenile salamanders as bait is known to have contributed to the spread of ranavirus in ambystomatid salamanders [25,26], which appears to represent one incidence in a long history of human introductions of pathogens to naive populations [27]. Here, we evaluate the relative importance of human-mediated spread versus other possible explanations for the apparent rapid recent spread of ranavirus within the UK.

There is now a considerable body of research on climate change as a driver of EIDs with documented effects on both hosts and pathogens [28,29]. Climate change can alter amphibian behaviour such as timing and duration of hibernation, which may affect pathogen transmission opportunities [30,31]. Ranaviruses exhibit temperature sensitivity both in the wild and in the laboratory. Ranavirus replication is more rapid at higher temperatures when grown under controlled conditions in cell culture [32]. In an animal model, experimentally infected common frog tadpoles experience higher mortality rates at higher temperature [33]. In the wild in the UK, ranavirus outbreaks show seasonality with a summer peak [34]. Although it is problematic to extrapolate directly from laboratory studies to ecology in the wild, such results indicate how climate change could alter the spatial distribution of ranavirosis.

Mapping of suspected ranavirosis events has consistently yielded a picture of apparent spread across England but has not previously accounted for reporting effort or considered other potential biases in the data. We address these problems and reconstruct spread using epidemiological models to assess whether classical epidemic spread, spatio-temporal patterns in an environmental variable predicted to affect host–pathogen interactions, or human behaviour better predict the emergence of the disease. We then combined this analysis with complementary information from an analysis of pathogen genotypes to reconstruct the pattern of ranavirus emergence.

## 2. Material and methods

### (a) Citizen science surveillance: The Frog Mortality Project

The Frog Mortality Project (FMP) collated reports of amphibian mortality from the public between 1992 and 2013 before it was subsumed into the Garden Wildlife Health project [35]. Methods used to seek and administer reports changed somewhat over this period (full details are provided in the electronic supplementary material). Steps taken as part of this study to prepare the FMP relational database for downstream analyses (particularly the georeference and time data) are also detailed in the electronic supplementary material.

The reports were filtered for consistency with ranavirus infection. ‘Ulceration’, ‘red spots on the body’, and ‘limb necrosis/loss of digits’ were the signs of disease chosen to reliably represent ranavirosis [15,34]. Cunningham [34] showed a strong association between these signs of disease (as recorded in reports of citizen scientists) and additional signs of disease at autopsy as well as the presence of ranavirus in affected tissues [34]. From 1992 to 1996, 95 carcases were examined from 24 sites of wild amphibian mortality at which lesions consistent with ranavirosis were reported, and 19 carcases from three sites with no such reports. Ranavirus was detected in at least one carcase (using virus culture) from 23 of the 24 sites with lesions reported, but none of the others.

A similar approach to filtering the FMP database for ranavirus-consistent mortality events has previously shown that reporters' observations of these signs can be used as a reliable predictor of ranavirus occurrence [16]. As well as reports of lesions, we required mortality events to include at least five animals, in order to be classified as consistent with ranavirus infection. This rule makes use of the known virulence and infectivity of the virus [15,16] and replaced summer incidence, which had been used as a criterion by Teacher *et al.* [16]. This change was made because recent studies identified incidents of ranavirus aetiology (confirmed by molecular methods) outside summer—between March and October [36]. All remaining reports in the database were classed as negative.

### (b) Covariate data

The values of covariates were obtained at the resolution of districts, boroughs, and unitary authorities for England and Wales. Ordnance Survey Boundary Line data were obtained from the Edina Digimap service under OS OpenData license [37]. Monthly averages of climatic variables for all UK 5 × 5 km grid squares covering the study period (1991–2010) were downloaded from the Met Office UKCP09 dataset. Regional human population densities were obtained from the Population Estimates Unit of the Office for National Statistics. Covariates were decomposed by year and by region (additional details of these methods are provided in the electronic supplementary materials). Climatic variables were strongly correlated (Pearson's correlation coefficients range from 0.46 to 0.92, see the electronic supplementary material, figure S1). We considered mean daily maximum temperature the most suitable climatic covariate, given the apparent peak incidence of ranavirosis in summertime, and correlations between temperature and virus growth in cell culture. Temporal and spatial patterns in population density and maximum temperature were visualized and analysed using linear regression in R.

### (c) Two-component spatio-temporal models

We used *twinstim*, a function in the R package Surveillance v. 1.7 [38–40], to analyse the UK spread of ranavirus-consistent mortality events. Outbreaks were modelled as Poisson events. The conditional intensity function (CIF) is the instantaneous rate or hazard for events at time, *t*, and location, *s*, conditioned on the history of all observations up to time, *t* [39]. The CIF is formulated as the sum of two components—the ‘endemic’ and ‘epidemic’ components (*h*() and e*() in the following equation):2.1
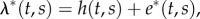
where *λ*() is the function specifying a Poisson rate of infection. The definitions of the terms ‘endemic’ and ‘epidemic’ differ from classical epidemiological definitions.

The ‘epidemic’ component (function e*()) indicates the contribution to the infection rate due to transmission from existing outbreaks and can be thought of as spread from pond to pond mediated via amphibian dispersal. This is sometimes termed the ‘self-exciting’ component [38] and describes the infection pressure at a given time and location due to all other events in the history up to that point. ‘Interaction functions’ model the decay of infectivity with distance (‘spatial interaction function’, in the terminology of the R package) and time (‘temporal interaction function’) from the infection source [40].

The ‘endemic’ component (function *h*()) is used to characterize infections arising from sources outside of a conventional system of transmission; i.e. they do not originate from a historic infection but emerge—are ‘imported’—from outside of the transmission system. The function includes an offset, which we used to allow for the number of amphibian ponds ‘at risk’, having controlled for reporting effort (see ‘Controlling for reporting effort: estimating the at risk population’)—such that the endemic rate of infection is proportional to the relative number of ponds under surveillance occupied by susceptible amphibians.

We explored the evidence for two alternative hypotheses for the varying incidence of these ‘endemic’ cases of ranavirosis: human translocation of virus, modelled by using human population density as a covariate, and climate-change effects, modelled using temperature as a covariate (daily maximum temperature averaged across a calendar year).

### (d) Model parametrization

Upper limits for the infectivity of events were set based on the biology of frogs. We set the spatial limit for any pond to transmit infection by the movement of infected individuals at 30 km (based on [41]) and the temporal limit at 2 920 days (an estimate of the maximum lifespan of a wild common frog). We used human population density and daily maximum temperature averaged across a year as the variables in formulating the endemic components in the two competing models of spread. To benchmark the performance of our final model, we generated 500 unique covariate datasets by repeatedly randomizing region to the remaining data and ran the two-component population density model with these datasets as input.

Goodness of fit was assessed using the method of Ogata and implemented as part of the Surveillance R package [39,42]. We also evaluated the model using simulated outbreaks based on the fitted parameter values. We ran 100 simulations from the fitted model without providing any data as ‘pre-history’ and simulations with some pre-history. We compared the mean total number of events and their spatial distribution to the real data. The real counts were assessed against the 2.5% and 97.5% quantiles of 100 realizations of the simulated model for each region [39].

### (e) Controlling for reporting effort: estimating the at risk population

**Figure 1.**
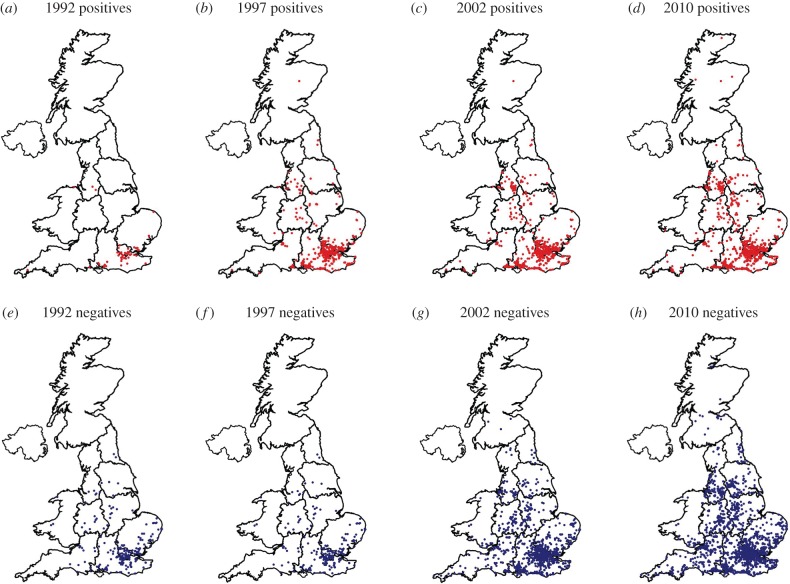
Visualization of UK ranavirus-consistent mortality events in time (1992–2010) and space (*a–d*) and non-consistent frog mortality reports for the same period (*e–h*). (Online version in colour.)

Reporting effort (number of citizen scientists recruited and the records they generated) varied across years and regions. In addition, the density of populations at risk (ponds used by susceptible common frog populations) also varied between regions. We reasoned that all of these issues could be allowed for by using the number of reports of mortality events that were negative (not ranavirus consistent; mapped in figure 1). The number of these records would increase in proportion to the reporting effort, and would be proportional to the number of ponds in any one locality. Their number, Nn, was therefore included as an offset (log-transformed since the Poisson model of events has a log link-function).

It is possible that some reporting biases are not compensated for in this manner, for example, if the relative rates of positive and negative reports were altered by the filtering of reporters or by appeals in the media to solicit public participation. One such large drive took place in London and the South-East in the early 1990s, and there have been other local and national media campaigns [34]. Of particular concern is the possibility that any association of outbreak incidence with human population density is actually a reflection of reporting bias, which has not been captured by our offset, since areas of high human population density are likely to have more reports. This issue was investigated by noting that a reporting bias effect would affect both ‘endemic’ and ‘epidemic’ components of the model, whereas long-distance translocations by humans (with an incidence proportional to human population density) would only affect the endemic component. We therefore compared the model with human population density included as a covariate in the ‘endemic’ component with one in which it was included in both components.

### (f) Phylogenetics

Nucleotide sequences downloaded from the National Center for Biotechnology Information (NCBI) nucleotide database (listed in the legend of figure 5) were aligned to sequence data from seven UK ranaviruses [43], using BLAST to pull out homologous regions. Sequence alignment and phylogenetic tree construction followed Price *et al.* [8]. There are no DNA sequence-derived estimates of substitution rates for ranaviruses, but rates across dsDNA viruses are thought to range from 10^−5^ to 10^−8^ substitutions per site per year (e.g. [44]). We used the upper limit to calculate a maximum-likelihood estimate of the minimum time to the most recent common ancestor of the UK viruses (R script provided; see the electronic supplementary material, Appendix S1). Support limits were calculated by taking values corresponding to two log-likelihood units either side of the maximum-likelihood estimate [45].

## 3. Results

After purging the citizen science database of records with essential data missing and obvious errors, a total of 4 460 reports remained. Filtering the database for reports consistent with ranavirosis in England and Wales produced a ‘positive’ set of 1 446 (32% of the total). Report numbers—both positive and total—were concentrated in particular years (see the electronic supplementary material, figure S2) and regions (e.g. 11% of total reports were received in 1995 from southeast England). Report data are visualized in space in figure 1, which shows a time series of the changing distribution of reports—both those consistent with ranavirosis (positive) and those that are not (negative). Both types of report accumulated over time and their geographical distribution increased, although the pattern was broadly similar for both types. It is not clear from these figures whether ranavirosis has spread or whether reporting effort has driven the change in distribution.

### (a) Spatial and temporal variation in main covariates

**Figure 2.**
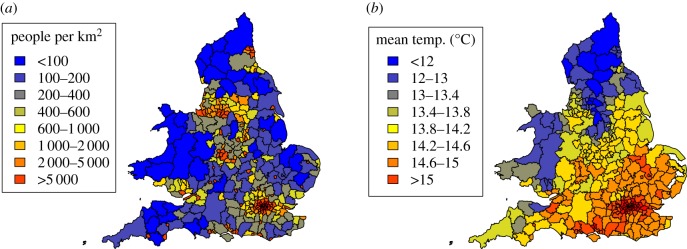
Regional variation in main covariates averaged across study period, 1991–2010 (*a*) human population density (people per square kilometre) and (*b*) mean maximum daily temperature (°C).

When we examined regional patterns in the variables associated with our hypotheses (human population density and temperature) we revealed some correlations, which can be visualized on maps (figure 2). For example, London was both warmer and much more densely populated than other regions, whilst Wales and parts of northern England were cooler and more sparsely populated. However, there were sufficient differences to discriminate between the two datasets; in particular, temperature decreased in a fairly consistent wave-like fashion from southeast England to Wales and northern England, whereas variation in population density was more of a mosaic. In addition, there were differences in the trends over time: mean daily maximum temperature across years increased in nearly all study regions, with the majority increasing by 0.5–0.8°C (inter-quartile range was 0.58–0.73°C; electronic supplementary material, figure S3), and there was a larger degree of change in the cooler north. Upward trends in population density showed more variation between regions (electronic supplementary material, figure S3) with a greater degree of change in the south.

### (b) Model outputs and performance

Human population density models consistently outperformed models with regional temperature as a covariate. The human population density models had higher likelihood and lower Akaike information criterion (AIC) scores than the temperature models (table 1). When fitting simple endemic models (excluding pond-to-pond infection), both these covariates were significant terms. For more biologically realistic ‘self-exciting’ models (including pond-to-pond infections via amphibian dispersal), temperature was no longer a significant term in models that also contained population density (*p* = 0.26). The model including only human population density (AIC = 33 072, logLik = −16 529), with dispersal between ponds modelled with a power-law function, performed better than the equivalent temperature only model (AIC = 33 279, logLik = −16 633). Human population density was a highly significant term (*p* < 2 × 10^−16^). By contrast, in the comparable temperature model, temperature was a non-significant term (*p* = 0.97). When human population density was included in both ‘endemic’ and ‘epidemic’ components—as a test of whether the correlation with human density was a reporting effort effect—model performance was not improved (AIC = 33 074, logLik = −16 529) and population density on the epidemic side was a non-significant term (*p* = 0.87).

**Table 1.** Spatio-temporal model summaries (two-component models with power-law spatial interaction function or endemic component only) for each of the endemic covariates. All models include the number of ‘negative’ records (see text) as an offset to control for reporting effort and represent the ‘population at risk’.

model class	log-likelihood	AIC	endemic covariate	*p*-value	coefficient
**two-component**					
population density	−16 529	33 072	pop. density	<2 × 10^−16^	4.89 × 10^−4^
temperature	−16 633	33 279	av. max. temp	0.97	4.17 × 10^−3^
population density + temperature	−16 526	33 068	pop. density av. max. temp	<2 × 10^−16^ 0.26	4.47 × 10^−4^ 9.95 × 10^−2^
population density in both components	−16 529	33 074	endemic pop. density epidemic pop. density	<2 × 10^−16^ 0.87	4.95 × 10^−4^ −4.58 × 10^−6^
population density × free school meals	−16 452	32 922	pop. density free school meals pop. density × free school meals	<2 × 10^−16^ <0.0005 6 × 10^−14^	1.74 × 10^−3^ 1.33 × 10^−1^ −9.23 × 10^−5^
**endemic only**					
population density	−18 952	37 910	pop. density	<2 × 10^−16^	4.58 × 10^−4^
temperature	−19 138	38 283	av. max. temp	<2 × 10^−16^	9.92 × 10^−1^
population density + temperature	−18 766	37 540	pop. density av. max. temp	<2 × 10^−16^ <2 × 10^−16^	3.70 × 10^−4^ 6.47 × 10^−1^
population density × free school meals	−18 689	37 388	pop. density free school meals pop. density × free school meals	<2 × 10^−16^ 7.81 × 10^−9^ <2 × 10^−16^	1.31 × 10^−3^ 6.69 × 10^−2^ −5.85 × 10^−5^

In both population density and temperature models, the ‘endemic’ component – which models the occurrence of ‘imported’ events—explained most events at the outset of the time series. Once such initial infections were established, the estimates of transmission from pre-existing infections predominated: in the two-component models the ‘endemic’ proportion fell below 20% within 5 years and remained fairly stable from then on. The estimates of ‘infectivity’ declined rapidly with distance, with low rates over distances in excess of 2 km. The power-law dispersal functions were almost identical between temperature and population density models. Both models had a residual distribution consistent with the fitted Poisson CIF (see Material and methods and electronic supplementary material, figure S4).

**Figure 3.**
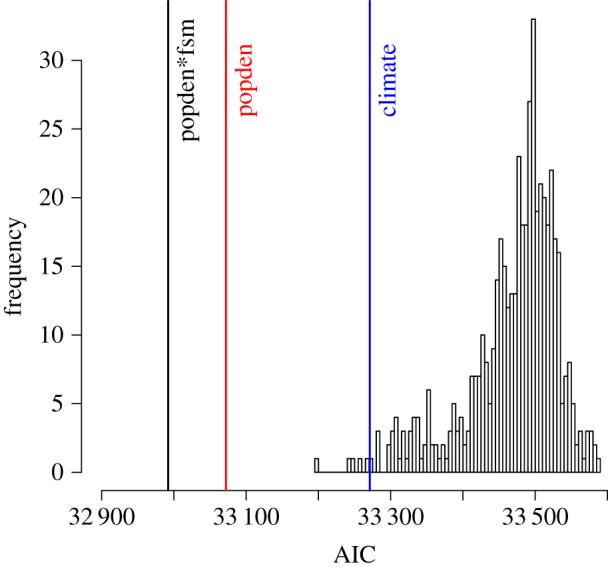
Comparison of model performance assessed by Akaike information criterion (AIC). Models featuring main covariates—population density (vertical line marked ‘popden’) and mean maximum daily temperature (vertical line marked ‘climate’)—are compared with the extension of the population density model including the interaction with the proportion of school students receiving free school meals (vertical line marked ‘popden*fsm’). All models are compared to 500 iterations of the population density model where each iteration used a unique randomization of region to the population density data as input (bars of histogram). (Online version in colour.)

**Figure 4.**
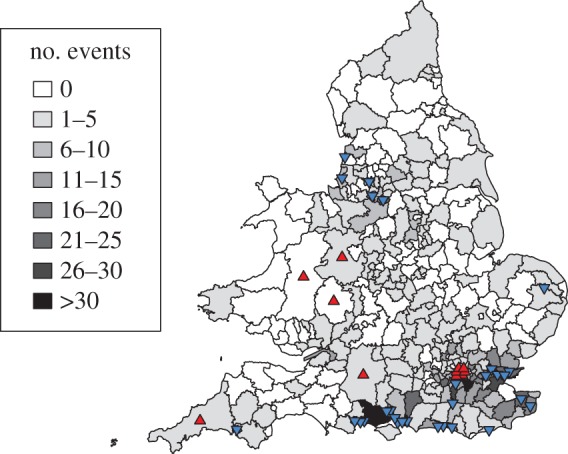
Comparison of spatial point pattern for real versus simulated data for 100 simulations from the fitted population density model with no data provided as pre-history. Intensity of shading represents the number of observations of ranavirus outbreaks in the region. Triangles indicate regions where simulations overestimated (red triangle points up) or underestimated (blue triangle points down) the real data. Regions where the real data fall inside 95% range of 100 realizations of the simulated model have no triangle. (Online version in colour.)

The population density model with the real data as input performed very well (lower AIC and higher log-likelihood scores) when benchmarked against 500 iterations using unique input datasets where region was repeatedly randomized to the remaining data (figure 3). By contrast, the temperature model performance was similar to the randomized datasets. We also ran simulations from the two-component population density model as another measure of its performance. The total number of events in the real data was 1 446 and simulations did well in matching this with a mean of 1 538 without any data being provided as ‘pre-history’ of outbreak locations. The model also performed well in predicting where new events occurred, with the number of simulated events matching the real data well for most regions (figure 4). Exceptions were the southeast and the northwest where, in the absence of ‘pre-history’ data, simulations underestimated numbers given the high numbers of reports originating from these areas in the early years of data collection.

In London, the simulations predicted more events than were actually observed, which may be a consequence of the much higher proportion of people living in apartment blocks (with less access to ponds) compared with other regions of the UK (UK Census Data, Office for National Statistics). To explore this hypothesis, we extended the two-component population density model to include an interaction term between population density and the regional proportion of school students receiving free school meals (a widely used proxy measure of socio-economic status in the UK). We hypothesized that this variable would be highly correlated with the proportion of people without access to a garden and negatively correlated with the overall amount of green space in a region. We found that the inclusion of this interaction did indeed improve the model fit (AIC = 32 922, logLik = −16 452; figure 3 and table 1) as well as the match between the number of real and simulated events (1 446 and 1 505, respectively) and the number of regions where simulations matched the real data (electronic supplementary material, figure S5).

### (c) UK *Ranavirus* diversity revealed by virus phylogenetics

**Figure 5.**
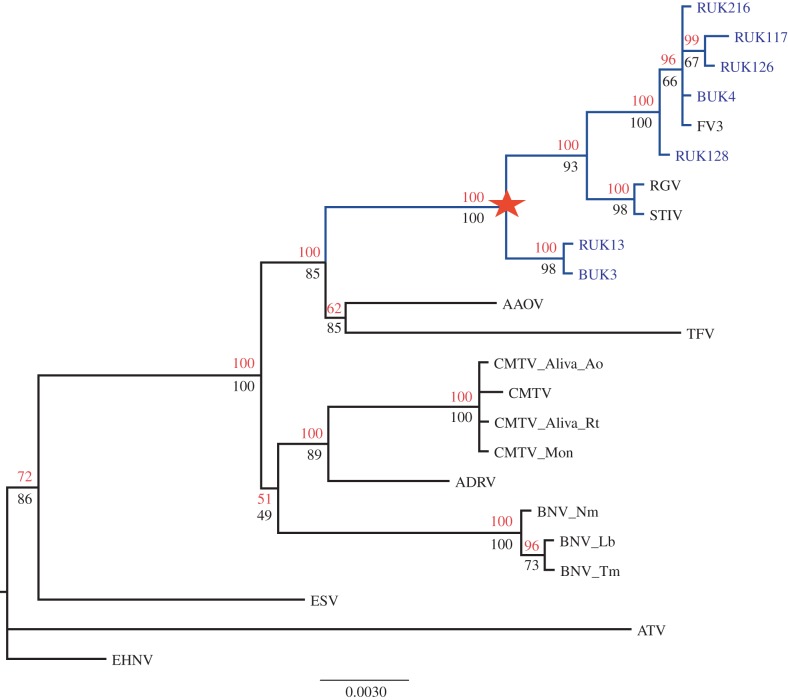
UK *Ranavirus* diversity in a global context. Monophyly of UK ranaviruses requires inclusion of Chinese and North American viruses. The tree was constructed from seven concatenated multiple sequence alignments [8]. Node support values are annotated on the Bayesian tree and calculated using maximum likelihood (bootstraps, bottom) and Bayesian inference (posterior probabilities, top) under a GTR model of molecular evolution. Scale of branch lengths is in nucleotide substitutions/site. UK viruses are labelled in blue and labels start ‘RUK’ or ‘BUK’. Additional sequences included are *Frog virus 3* (FV3, AY548484), Tiger frog virus (TFV, AF389451), *Ambystoma tigrinum virus* (ATV, AY150217), *Epizootic hematopoietic necrosis virus* (EHNV, FJ433873), Soft-shelled turtle iridovirus (STIV, NC012637), Rana grylio virus (RGV, JQ654586), European sheatfish virus (ESV, JQ724856), Chinese giant salamander virus (ADRV, KC865735), Common midwife toad virus (CMTV, JQ231222), Bosca's newt virus (accession numbers for individual loci as [8]). (Online version in colour.)

Our final multiple sequence alignment contained 2 267 base pairs from 23 virus isolates (seven from the UK and 16 viruses from elsewhere), which we used to reconstruct a ranavirus phylogeny. The overall topology inferred by both Bayesian and maximum-likelihood methods was identical. UK viruses formed two groups with RUK13 and BUK3 forming an outgroup clade (figure 5). Monophyly of all UK ranaviruses was not supported. Time to the most recent common ancestor of UK viruses (the node marked with a red star in figure 5) was estimated at 332 years ago (95% CI = 189–533 years ago) assuming a substitution rate of 10^−5^ subs site^−1^ yr^−1^.

## 4. Discussion

We used data generated by a citizen science surveillance project in combination with occasional genetic sampling to reconstruct emergence of an important wildlife pathogen. By controlling for reporting effort and applying spatio-temporal modelling techniques, we have overcome the limitations of common epidemiological techniques, such as cluster analyses. These approaches have dealt with our concern that the apparent geographical spread of UK ranavirus events might be an artefact of reporting effort.

The use of an ‘epidemic’ component in models and our finding that a high proportion of events were attributed to it showed that the majority of reported incidences of ranavirosis are likely to have arisen via transmission from nearby ponds and we recovered estimates of dispersal consistent with the known ecology of frogs. This type of viral transmission would have created a classical wave-like spread, with a timescale and spatial pattern that explains much of the observed data. In addition, there was a small but significant proportion of events that were explained by ‘endemic’ processes which model other sources of infection including infection from non-local sources. We explicitly controlled for reporting effort by including the number of reports of frog mortality not consistent with ranavirosis as an offset in our models. In this way, the analysis answered the question ‘where are the ponds with human observers?’ and forced the infection rate to be proportionate to this variable. Over and above this observer bias, the pattern of new outbreaks was strongly predicted by human population density; we have interpreted this pattern as evidence for the translocation of infectious materials by people, enhancing the spread of a novel pathogen over greater geographical distances at shorter timescales than could be accomplished through typical frog movements.

The modelling process was correlational so requires the usual caveats of such studies—it is not possible to completely rule out some other influence of human population density on the outcome—for example, environmental pollutants could have amplified the effects of pre-existing ranavirus infections that had previously gone undetected. However, such hypotheses would require the virus to have been widespread already. Since we have shown that the majority of recorded events can be explained via transmission between nearby ponds over the previous two decades, human translocations of infectious materials over a similar time period seems a more parsimonious explanation for the ‘endemic’ contribution to the spread. The further improvement of the population density model following inclusion of the interaction with a measure of socio-economic status also adds support to this interpretation: there is a predictable effect of this additional covariate on access to ponds but it is more difficult to envisage how this interaction would modify a correlation between human population density and the detection of disease.

We used phylogenetic analysis based on some limited sampling of infected tissues as a complementary approach to the spatio-temporal models and found support for dispersal through human translocations when interpreted in combination with the modelling. Using our conservative estimate of the minimum time to the most recent common ancestor, it is clear that the genotypic diversity in UK viruses cannot have arisen during the course of its spread over the last 25–30 years.

Hyatt *et al.* [22] previously obtained phylogenetic data suggesting an introduction of ranavirus to the UK from North America, possibly via the pet trade. In this context, our new phylogeny suggests that there have been at least two introductions, each with a distinct history. It is likely that further analysis of samples taken across the geographical distribution in figure 1 would identify other translocations including long-distance transfers within the UK that facilitated emergence. Previous work has identified several possible sources of such ranavirus introductions, by polymerase chain reaction (PCR) screening of animals that are traded, cultured, and invasive (e.g. North American bullfrogs, which have escaped from farms and the pet trade) [17–21,46,47]. Human translocations of infected animals have driven ranavirus emergence on a broad scale, for example, through the use of infected salamanders as angling bait in North America [25,26]. Prevalence of ranavirus infection is associated with human activity in Canada and previous work has shown occurrence of ranavirosis in the UK to be associated with urban environments [23,24]. Some of the international spread of ranavirus may be associated with the global trade in animals [17,48]. This trade is huge in magnitude: for example, nearly 38 million animals from 163 countries were imported to the USA in a 5-year period at the turn of the century and 51 species of non-native amphibians and reptiles have been recorded in Greater London since the 1980s [49–51].

## 5. Conclusion

Our results suggest further lines of research to help control the spread of ranavirus infections in the UK. Daszak *et al.* [9] identified two broad categories of human intervention affecting the emergence of infectious diseases of wildlife which should be investigated: spread (i) by spillover of infection from domestic animals and (ii) by human translocations of pathogen or host. The first could be corroborated by the identification of a vector; fish or non-native amphibians (e.g. North American bullfrog) being candidates for reservoir hosts [19,52]. The second category could involve the translocation of fomites, such as aquatic plants, or infected animals, e.g. spawn, tadpoles, or frogs. Targeted sampling of such potential vectors, plus further genetic sampling of ranaviruses to gain a more complete picture of pathogen diversity would further address the mode and scale of translocations. In the meantime, existing recommendations discouraging the movement of vectors and fomites could be much better publicized as an interim step.

This study also represents an important general contribution to the field of emerging wildlife disease through the demonstration of the potential and applicability of its methodological approach. Our methods have enabled reconstruction of ongoing disease emergence in a timescale enabling the information to flow into management decisions. This approach can be more widely useful when working with a pathogen where the mutation rate, biology, and practicalities of sampling reduce the utility of fashionable phylodynamic techniques, which are more appropriate for fast evolving and intensively sampled RNA viruses. Emergent disease risks are posed by all types of pathogen, many of which, like ranaviruses (DNA viruses), likely have lower mutation rates.

Although awareness of the ongoing biodiversity crisis has increased and is a clear and strong motivation for assembling comprehensive datasets, wildlife disease remains poorly represented compared with disease affecting humans and domestic animals. The approach used here, which builds on a citizen science surveillance project in combination with mainly opportunistic genetic sampling, therefore, represents a promising approach for the reconstruction of emerging wildlife diseases and exploration of hypotheses that can inform conservation strategies. To this end, we hope this study will encourage others to both generate additional datasets of this type (following initiatives like the Garden Wildlife Health project, http://www.gardenwildlifehealth.org) and to apply the same approach to existing data.

## Supplementary Material

Electronic Supplementary Material

## Data accessibility

Citizen science database used in models and nucleotide sequence alignment used in phylogeny construction are available in Data Dryad: http://dx.doi.org/10.5061/dryad.27c8s [43].
