# Supplementary material for: Reconstructing the emergence of a lethal infectious disease of wildlife supports a key role for spread through translocations by humans
Source: Proc Biol Sci. 2016 Sep 28;283(1839):20160952. doi: 10.1098/rspb.2016.0952 (PMC5046891; doi:10.1098/rspb.2016.0952)
Supplement: Electronic Supplementary Material [file rspb20160952supp1.pdf]

Electronic Supplementary Material

Proceedings of The Royal Society B: Biological Sciences

Reconstructing the emergence of a lethal infectious disease of wildlife supports a key role for spread through translocations by humans

Stephen J. Price, Trenton W.J. Garner, Andrew A. Cunningham, Tom E.S. Langton, Richard A. Nichols

Supplementary methods

The Frog Mortality Project (FMP) database: Reports were solicited through nationwide appeals via all forms of the popular media - annually until 1997 and intermittently since then (1). Records were maintained as hard copies until 2001 and a mixture of electronic and paper thereafter. The current study is the first analysis of the pattern of UK ranavirus spread using the complete FMP database produced after a major round of retrospective data entry of paper-based records (funded by Natural England and administered by Froglife). Prior to 2001, paper reports were issued on request, usually after correspondence about the nature of the mortality events; hence, those that did not immediately appear to be consistent with ranavirosis were sometimes not pursued further. After this, reports were increasingly filed through an unrestricted online form. The change in the method of data acquisition may therefore have changed the frequency of reports and the overall proportion of submitted reports not relating to ranavirosis.

Preparation of the FMP database for analyses: Postcodes were submitted with reports and we used them to provide approximate geographical positions for mortality events. Although postcodes are discrete data, they cover relatively small areas (typically only 15 households) and could therefore be used to generate continuous spatial coordinates for this study. Each

postcode was converted to Ordnance Survey National Grid References (coordinate reference system, “EPSG, 27700”) using GeoConvert Postcode Data for 2006 onwards form the National Statistics Postcode Directories (http://geoconvert.mimas.ac.uk, accessed 10/09/14). The precision of timestamp information in the FMP database in relation to onset and duration of mortality events was usually at the resolution of month. There is some uncertainty about the timing of transmission and the onset of disease: ranavirosis outbreaks are focused in adults in the UK (2) and appear to have a peak incidence in summer months (1). However, transmission by direct contact during the breeding season with a lag in disease onset is a possible route in the wild (3), and other routes are possible – as indicated by the variety of exposure methods by which experimental ranavirus infections can be elicited (4). For these reasons we concluded that an outbreak could be classified to the year in which it occurred, but to no finer resolution. To break tied timestamps (a requirement of the modelling approach used), unique times for each event were generated by randomly drawing a time in days on a continuous scale from within the year of the report.

Covariate Data: In order to assign events to region, boundary coordinate data were simplified in Mapshaper v. 0.2.14 (5) using the default algorithm (Visvalingam simplification with modified smoothing) and read into R (6) as a shapefile. These regional polygons were then fused to give a spatial polygons object representing England and Wales. We used The European Terrestrial Reference System 1989 (EPSG 3035) in kilometre units as the coordinate reference system. The locations of ranavirus-consistent reports were converted to the same coordinate reference system and plotted to check if they fitted within the simplified boundary. Coordinates of the polygon were edited manually where points were located outside of the simplified boundary.

UK climate data were downloaded from the Met Office UKCP09 dataset and were processed

in R. We downloaded monthly averages of climatic variables for all UK 5x5km grid squares over our whole study period (1991-2010). The grid squares were overlaid on a map of the UK with boundaries marked in order to assign them to the appropriate region, prior to calculation of annual, regional averages from all 5x5km grid squares. The Met Office makes data available for a number of climatic variables, which are strongly correlated (Pearson's correlation coefficients range from 0.46 to 0.92, see Figure S1 in Supplementary material). We considered mean daily maximum temperature the most suitable variable to use in models given the apparent peak incidence of ranavirosis in summertime, which could be indicative of some temperature-related effect, as well as increasing temperature yielding increased virus growth in cell culture (7). Linear models of the maximum daily temperature averaged by year and region were fitted and were analysed in R. Regional human population densities were obtained from the Population Estimates Unit of the Office for National Statistics. As with the climatic covariate, temporal and spatial patterns in population density were visualised and analysed using linear regression in R.

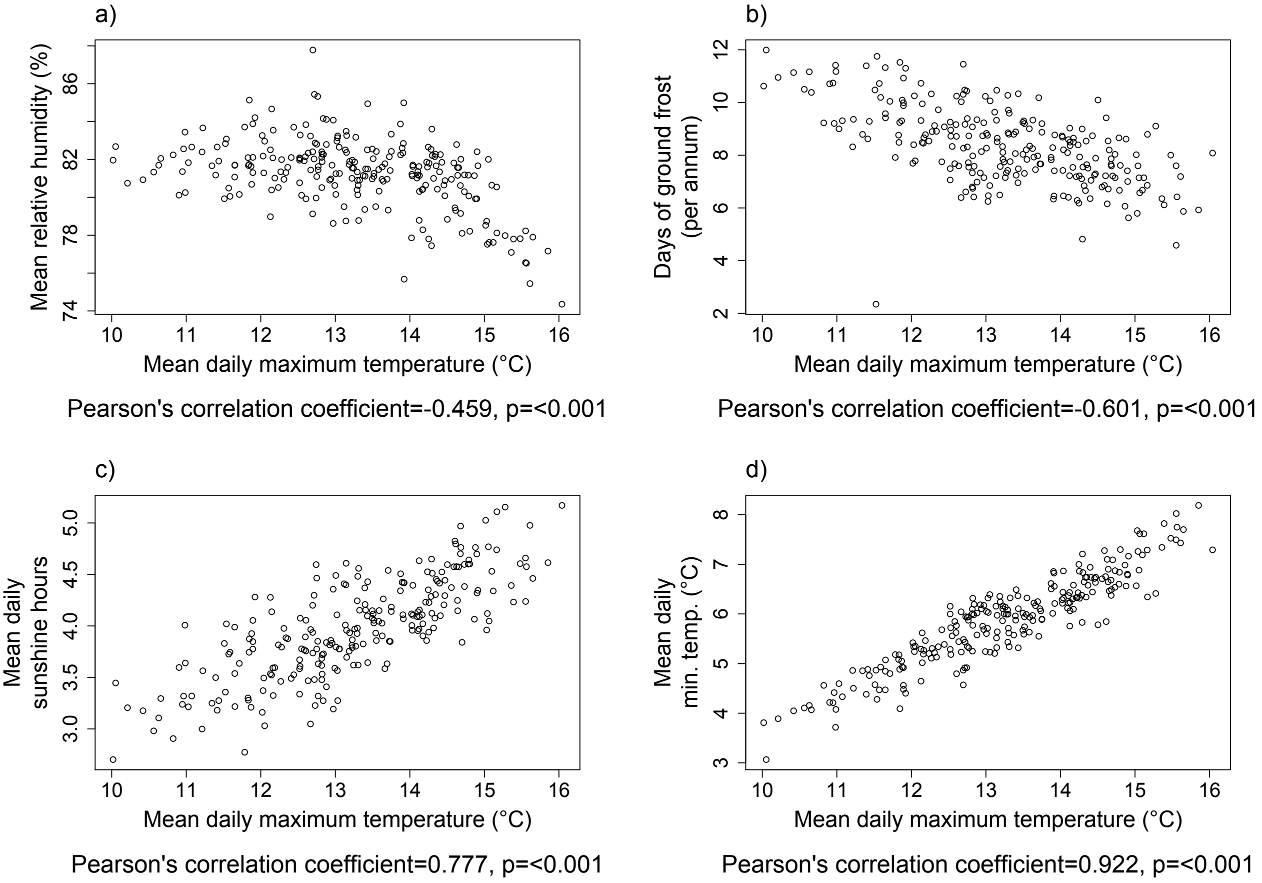

Figure S1. Correlation between mean daily maximum temperature and other UK Met Office UKCP09 climatic variables. Plots show a) humidity, b) ground frost, c) hours of sunshine, d) daily minimum temperature against mean daily maximum temperature for study area between 1991 and 2010. Correlation coefficients and p-values (Pearson's product-moment correlation calculated in R) are included below plots.

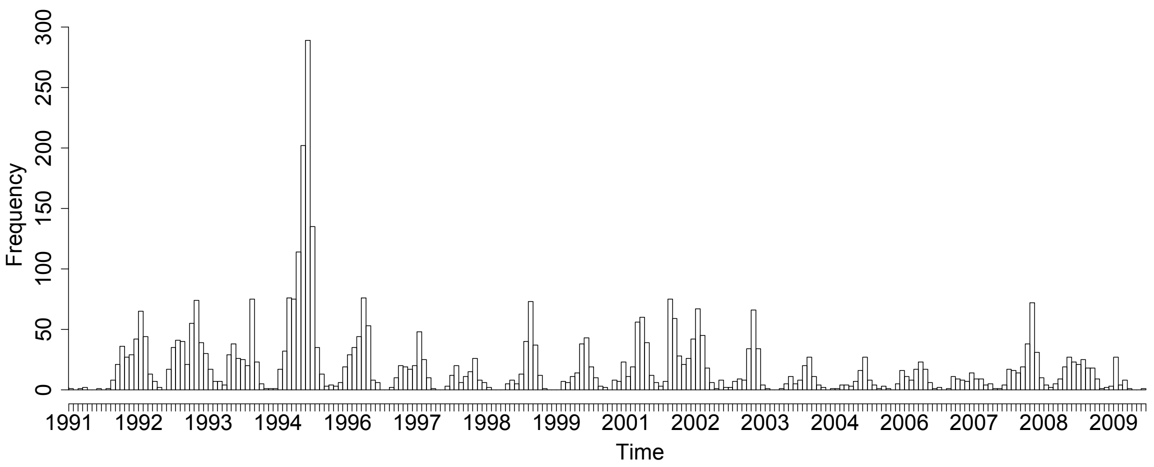

Figure S2. Number of reports of frog mortality received by month by the Frog Mortality Project for the period 1991-2010.

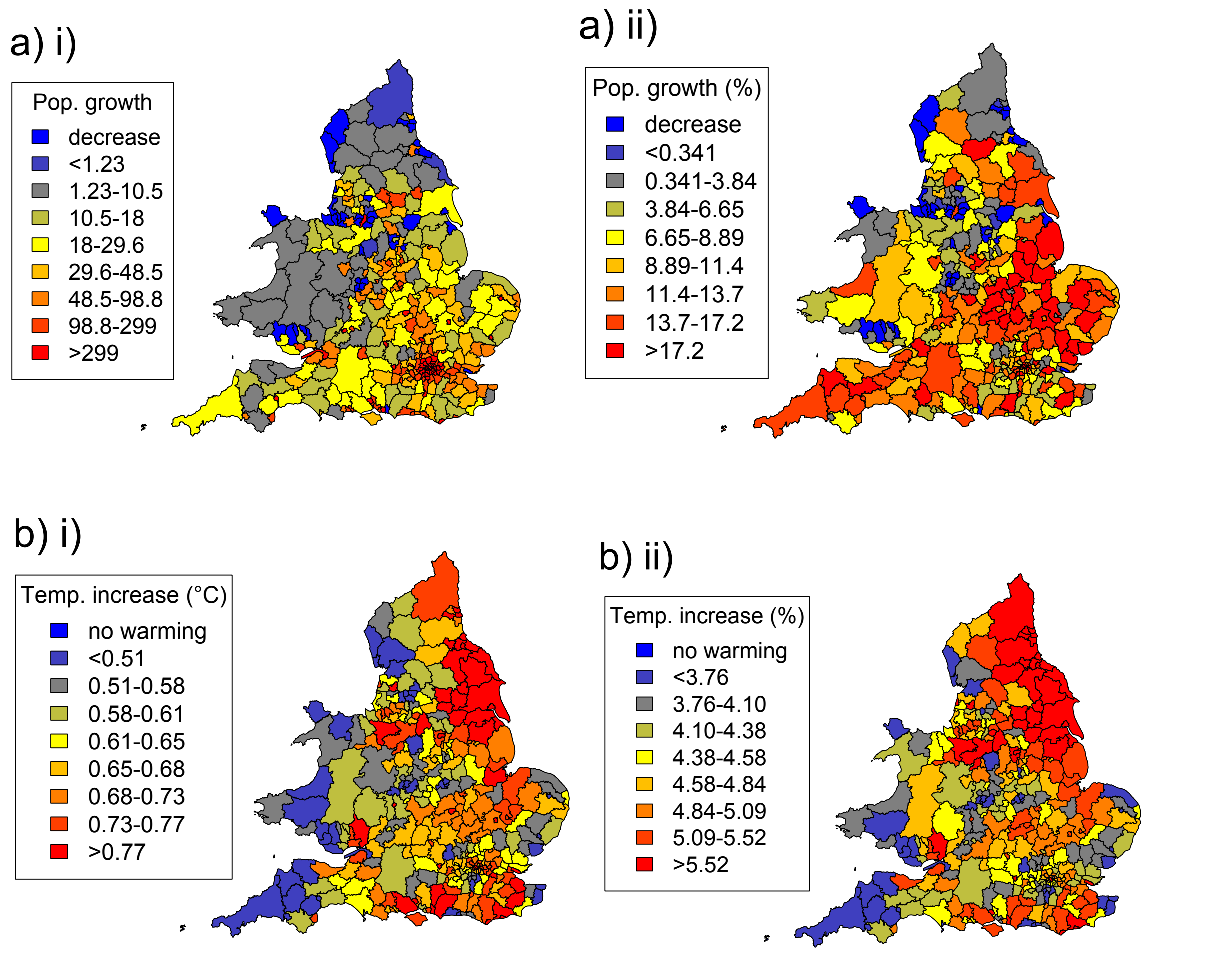

Figure S3. Summary of temporal trends in population density and temperature by region. a) regional population density, b) mean regional daily maximum temperature. Left-hand plots show overall difference between 2010 and 1991 as predicted by simple linear model. Right-hand plots show this predicted difference standardized by the local mean value for the period 1991-2010 to give change in percentage terms.

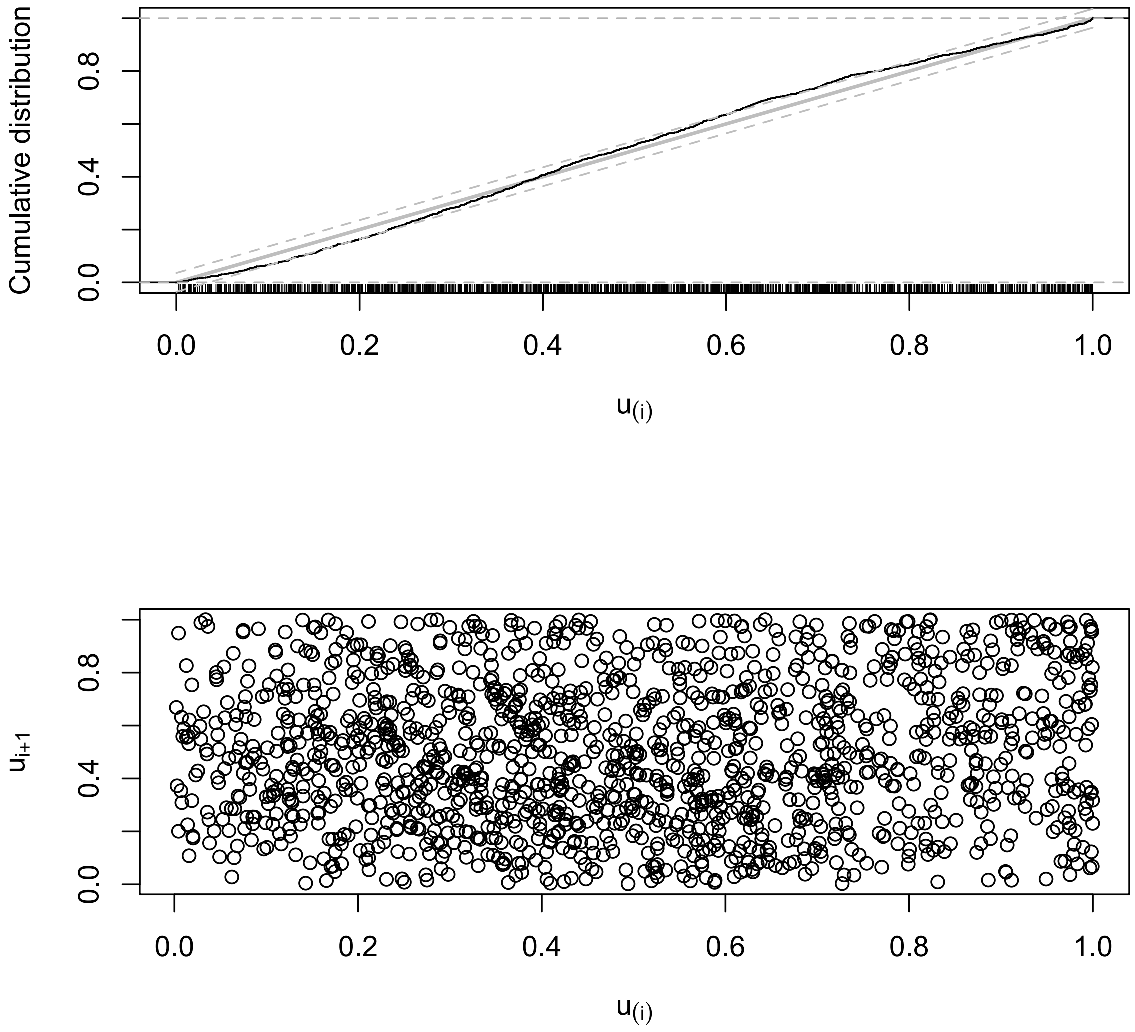

Figure S4. Assessment of goodness of fit of population density model through examination of the residual process – transformed time [$u_{(i)}$] against the cumulative distribution of the conditional intensity function (CIF) after Ogata (1988).

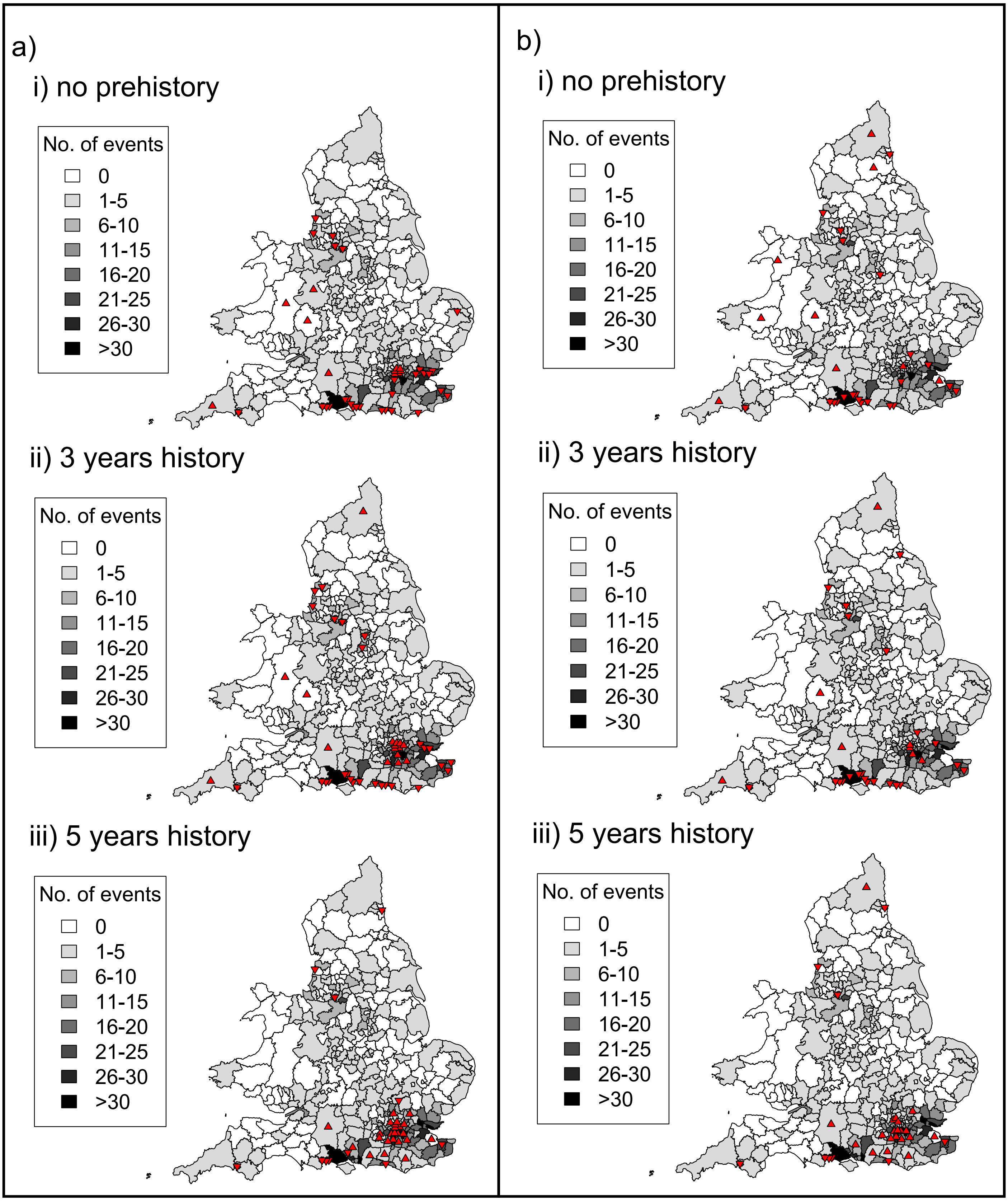

Figure S5. Comparison of model simulations with no data provided as prehistory to those with 3 years history and 5 years history. a) population density model, b) population density with free school meals interaction.

Appendix S1. R Script to calculate maximum likelihood estimate of lower bound on time to the most recent common ancestor of UK ranaviruses.

```
# Script to calculate the MLE and support limits on number of
 years since a common ancestor given a mutation rate
# and the observation of 15 mutations out of 2269 sites


like13b<-function(u,years) dbinom(15,2269,1-(1-u)^(years*2),T)

Nyears<-1:1500

plot(Nyears,like13b(10^-5,Nyears),type='l')

abline(h=max(like13b(10^-5,Nyears))-2)

hiRange<-which(like13b(10^-5,Nyears)>(max(like13b(10^-
 5,Nyears))-2))

supportLims<-c(hiRange[1],hiRange[length(hiRange)])

maxL<-which(like13b(10^-5,Nyears)==(max(like13b(10^-
 5,Nyears))))

abline(v=supportLims)
abline(v=maxL,col='red')

supportLims
maxL
```

Supplementary References

1. Cunningham AA. Investigations into mass mortalities of the common frog (Rana temporaria) in Britain: epidemiology and aetiology [Internet]. Royal Veterinary College (University of London); 2001 [cited 2013 May 9]. Available from: http://ethos.bl.uk/OrderDetails.do?uin=uk.bl.ethos.269010

2. Duffus ALJ, Nichols RA, Garner TWJ. Experimental evidence in support of single host maintenance of a multihost pathogen. Ecosphere. 2014 Nov 1;5(11):142.

3. Brunner JL, Schock DM, Collins JP. Transmission dynamics of the amphibian ranavirus Ambystoma tigrinum virus. Dis Aquat Organ. 2007 Sep 14;77(2):87–95.

4. Cunningham AA, Hyatt AD, Russell P, Bennett PM. Emerging epidemic diseases of frogs in Britain are dependent on the source of ranavirus agent and the route of exposure. Epidemiol Infect. 2007 Oct;135(7):1200–12.

5. Harrower M, Bloch M. MapShaper.org: A Map Generalization Web Service. IEEE Comput Graph Appl. 2006;26(4):22–7.

6. R Core Team. R: A language and environment for statistical computing. R Foundation for Statistical Computing, Vienna, Austria. [Internet]. 2013. Available from: http://www.R-project.org/

7. Ariel E, Nicolajsen N, Christophersen M-B, Holopainen R, Tapiovaara H, Jensen BB. Propagation and isolation of ranaviruses in cell culture. Aquaculture. 2009 Sep 16;294(3-4):159–64.
